# 

**Published:** 1940

**Authors:** 


### The Medical Library of the University of Bristol

WITH WHICH IS INCORPORATED

**The Library of the Bristol Medico-Chirurgical Society**

---

*The following donations have been received since the publication of the list in July,* 1940.

November, 1940.

| | | |
|---|---|---|
| Messrs. John Bale & Staples, Ltd. (1) | .. .. | 1 volume. |
| Dr. C. C. Easterbrook (2) | .. .. .. .. | 1 volume. |
| Professor E. W. Hey Groves (3) | .. .. .. | 8 volumes. |
| Michell Clarke Fund (4) .. | .. .. .. .. | 3 volumes. |
| Munro Smith Fund (5) .. | .. .. .. .. | 1 volume. |
| Dr. J. A. Nixon (6) | .. .. .. .. .. | 2 volumes. |
| Royal College of Surgeons (7) .. | .. .. .. | 1 volume. |

Unbound periodicals have also been received from Dr. T. M. Ling, Dr. T. Milling, Professor C. Bruce Perry, Dr. G. de M. Rudolf, Dr. J. Odery Symes and Mr. E. Watson-Williams.

### THE ONE HUNDRED AND SEVENTY-SECOND LIST OF BOOKS

The figures in round brackets refer to the figures after the names of the donors The books to which no such figures have been attached have either been bought from the Library Fund or received through the *Journal.*

| | | |
|---|---|---|
| **Bateman, D.** .. .. | *Berkeley-Moynihan—Surgeon* .. .. .. .. (3) | 1940 |
| **Boyle, H. H.** .. .. | *Theory and Treatment of Fractures of the Jaws in Peace and War* .. .. .. .. .. .. .. (3) | 1940 |
| **Browne, F. J.** .. .. | *Advice to the Expectant Mother* .. 5th Ed. | 1940 |
| **Cameron, A. T.** .. .. | *Recent Advances in Endocrinology* 4th Ed. (4) | 1940 |
| **Clark, A. J.** .. .. .. | *Applied Pharmacology* .. .. .. 7th Ed. | 1940 |
| **Cunningham, D. J.** .. | *Manual of Practical Anatomy* 10th Ed. 3 vols. | 1940 |
| **Dargeon, H. W.** (Ed.) | *Cancer in Childhood* .. .. .. .. .. .. (3) | 1940 |
| **Dilling, W. J.** .. .. | *Pharmacology and Therapeutics of the Materia Medica* .. .. .. .. .. .. .. .. 16th Ed. | 1940 |
| **Easterbrook, C. C.** .. | *The Chronicle of Crichton Royal,* 1833–1936 (2) | 1940 |
| **Fearon, W. R.** .. .. | *An Introduction to Biochemistry* .. 2nd Ed. | 1940 |
| **Geckeler, E. O.** .. .. | *Fractures and Dislocations for Practitioners* 2nd Ed. (3) | 1940 |
| **Glover, E. G.** (Ed.) .. | *An Investigation of the Technique of Psycho-analysis* .. .. .. .. .. .. .. .. .. .. .. .. | 1940 |
| **Glover, E. G.** .. .. | *Psycho-analysis* .. .. .. .. .. .. .. .. .. | 1939 |
| **Grant, J. C. B.** .. .. | *A Method of Anatomy* .. .. .. 2nd Ed. (4) | 1940 |
| **Haldane, J. S.** .. .. | *Respiration* .. .. .. .. .. .. .. .. .. .. | 1927 |
| **Handfield-Jones, R. M.** | *Surgery of the Hand* .. .. .. .. .. .. (3) | 1940 |

| | | | |
|---|---|---|---|
| Harvey, W. C., and Hill, H. | *Insect Pests* | | 1940 |
| Hill, A. V. | *Muscular Movement in Man* | | 1927 |
| Homans, J. | *Circulatory Diseases of the Extremities* | | 1939 |
| Howell, W. H. | *Textbook of Physiology* | 14th Ed. | 1940 |
| Hutchison, R., and Hunter, D. | *Clinical Methods* | 11th Ed. (5) | 1940 |
| James, W. W., and Fickling, B. W. | *Injuries of the Jaws and Face with Special Reference to War Casualties* | (1) | 1940 |
| Jolly, D. W. | *Field Surgery in Total War* | (3) | 1940 |
| McCrea, E. D'A. | *Diseases of the Urethra and Penis* | | 1940 |
| McDougall, J. B. | *Tomography* | | 1940 |
| Manson, P. | *Tropical Diseases* | 11th Ed. | 1940 |
| Nightingale, F. | *Notes on Nursing* | (6) | 1860 |
| Oakes, L. | *Illustrations of Bandaging and First Aid* | | 1940 |
| Pye, W. | *Surgical Handicraft.* Ed. Hamilton Bailey | 12th Ed. | 1940 |
| Ross, J. A. | *Handbook of Radiography* | (3) | 1940 |
| Steindler, A. | *Orthopedic Operations* | (3) | 1940 |
| Walshe, F. M. R. | *Diseases of the Nervous System* | | 1940 |
| Worster-Drought, C. | *Neurosyphilis* | | 1940 |

TRANSACTIONS, REPORTS, JOURNALS, ETC.

| | | |
|---|---|---|
| Calendar of the Royal College of Surgeons, 1940 | (7) | 1940 |
| Collected Papers of the Mayo Clinic, Vol. XXXI | (4) | 1940 |
| Prospectuses of the Bristol Medical School, 1884–1894 | (7) | 1884–1894 |

### Publications Received

From MESSRS. BAILLIÈRE, TINDALL & COX:

*An Investigation of the Technique of Psycho-analysis.* Edited by EDWARD GLOVER, M.D. Price 10s. 6d.

From MESSRS. JOHN WRIGHT & SONS LTD.:

*The British Journal of Surgery.* No. 109. July, 1940. Price 12s. 6d.

*The British Journal of Surgery.* No. 110. October, 1940. Price 12s. 6d.

From MESSRS. JOHN BALE, SONS & STAPLES LTD.:

*Neurosyphilis.* By C. WORSTER-DROUGHT, M.A., M.D., F.R.C.P. Price 10s. 6d.

From DR. C. C. EASTERBROOK:

*The Chronicle of Crichton Royal* (1833–1936). By C. C. EASTERBROOK, M.A., M.D., F.R.C.P.

From MESSRS. HAMISH HAMILTON (MEDICAL BOOKS):

*A Textbook of Dietetics.* By L. S. P. DAVIDSON, B.A., M.D., F.R.C.P., F.R.S.E., and IAN A. ANDERSON, M.B., Ch.B. Price 10s. 6d.

From MESSRS. WILLIAM HEINEMANN (MEDICAL BOOKS) LTD.:

*The Art of Surgery.* By H. S. SOUTTAR, D.M., M.Ch., F.R.C.S. 4th Edition. 2 vols. Price 25s.

From MESSRS. H. K. LEWIS & CO. LTD.:

*Venereal Diseases.* By E. T. BURKE, D.S.O., M.B., Ch.B. Price 30s.

*Syphilis in Earlier Days.* By J. R. WHITWELL, M.B. Price 5s.

*Practical Orthoptics in the Treatment of Squint.* By KEITH LYLE, M.A., M.D., M.Ch., M.R.C.P., F.R.C.S., and SYLVIA JACKSON. 2nd Edition. Price 15s.

From MESSRS. E. & S. LIVINGSTONE :

*Diseases of the Nervous System.* By F. M. R. WALSHE, O.B.E., M.D., D.Sc., F.R.C.P. Price 12s. 6d.

*Surgery of Modern Warfare.* Edited by HAMILTON BAILEY, F.R.C.S. Part 1. Price 12s. 6d.

*Textbook of Medicine.* Edited by J. J. CONYBEARE, M.C., D.M., F.R.C.P. 5th Edition. Price 24s.

From OXFORD UNIVERSITY PRESS (HUMPHREY MILFORD) :

*Diagnosis and Treatment of Diseases of Peripheral Arteries.* By SAUL S. SAMUELS, A.M., M.D. 2nd Edition. Price 31s. 6d.

*Applied Physiology.* By SAMSON WRIGHT, M.D., F.R.C.P. 7th Edition. Price 25s.

*Malignant Disease and its Treatment by Radium.* By STANFORD CADE, F.R.C.S. Price 75s.

### Local Medical Notes

**Honorary Degrees.**—Professor Ernest Hey Groves is the recipient of the Honorary D.Sc. of the National University of Ireland, " in view of his eminence as an orthopædic surgeon and an internationally recognised authority on the literature of the subject."

EXAMINATION RESULTS.

**University of Bristol.**—Students of the University have recently passed the following examinations :—

M.B., CH.B.—*Supplementary First Examination. Pass :* D. C. Berry, A. C. Brown, C. T. Brown, M. J. Dunn, Pamela M. Farmer, Sheila E. Fraser, P. H. L. Hamilton, W. H. N. Moore, Joan G. McColl, Ann E. Read, Patricia M. Rundle, Rae H. Scott, A. D. Verniquet.

L.D.S.—*Supplementary First Examination. Pass :* A. R. Brook.

**Conjoint Board.**

L.R.C.P., M.R.C.S.—*First Examination. Pass : In Anatomy and Physiology :* Doris E. Counsell, Mildred I. Pott. *In Physiology :* J. T. N. Cole.

L.R.C.P., M.R.C.S.—*Final Examination. Pass : In Surgery :* J. H. G. Morris. *In Midwifery :* G. Schwizer, B. C. E. Aldous. *In Pathology :* A. G. Farr,* K. A. Hall.*

* Qualified.

### Index

Appointments, 68

Bombing of Bristol, 128
Book Reviews, 46, 99, 133
Bristol Royal Hospital.—New arrangements for out-patients, 67

Bristol University.—
Dental Hospital and School, 41
— Appointments, 68
Examination Results, 68, 105, 146
Medical Library, 62, 102, 144
British Red Cross Society in Bristol, 54
Burgess, N. F. C.—Obituary, 142

Cancer, Malum Immedicabile. — E. Watson-Williams, 9
Cerebro-Spinal Meningitis in Bristol.—B. A. Peters, 77

Davie, T. B.—Popular Misconceptions in Medical Matters, 107
Dunster, M., and Rendle Short, A.—Traumatic Extradural Hæmorrhage, Part I, 81

Editorial Notes, 54, 101, 141
Electro-Encephalography. — W. Grey Walter, 1

Glenny, E. T.—Obituary, 61
Gross, J. W.—Obituary, 58

Head Injuries in War-time.—F. W. Willway, 91

Hæmorrhage, Traumatic Extradural.—
Part I, A. Rendle Short and M. Dunster, 81
Part II, W. Grey Walter, 86

Induction of Labour by High Puncture of the Membranes.—C. Rendle Short, 121
Influenza (Editorial Note).—56

Long Fox Memorial Lecture.—Popular Misconceptions in Medical Matters. —T. B. Davie, 107

Middlesex Hospital (Editorial Note), 101
Midwifery in Bristol.—H. J. Drew Smythe and H. L. Shepherd, 117

Nuffield Provincial Hospitals Trust (Editorial Note).—141

Obituaries—
Julia Woolnough Gross, 58
George Henry Temple, 59
Elliott Thornton Glenny, 61
Norman Francis Clifford Burgess, 142

Peters, B. A.—Cerebro-Spinal Meningitis in Bristol, 77
Popular Misconceptions in Medical Matters (Twenty-ninth Long Fox Memorial Lecture).—T. B. Davie, 107

Save the Children Fund (Editorial Note).—142
Shelter Problem, 130

Shepherd, H. L., and Smythe, H. J. Drew. —Midwifery in Bristol, 117

Short, A. Rendle, and Dunster, M.—Traumatic Extradural Hæmorrhage, Part I, 81

Short, Rendle, C.—Induction of Labour by High Puncture of the Membranes, 121

Smythe, H. J. Drew, and Shepherd, H. L. —Midwifery in Bristol, 117

Temple, G. H.—Obituary, 59

Victoria Jubilee Convalescent Home.—New President (Editorial Note), 57

Walter, W. Grey.—
Electro-Encephalography, 1.
Traumatic Extradural Hæmorrhage, Part II, 86

Watson-Williams, E.—Malum Immedicabile Cancer, 9

Willway, F. W.—Head Injuries in War-time, 91

